# Chronic Chemogenetic Activation of the Superior Colliculus in Glaucomatous Mice: Local and Retrograde Molecular Signature

**DOI:** 10.3390/cells11111784

**Published:** 2022-05-29

**Authors:** Marie Claes, Emiel Geeraerts, Stéphane Plaisance, Stephanie Mentens, Chris Van den Haute, Lies De Groef, Lut Arckens, Lieve Moons

**Affiliations:** 1 Neural Circuit Development and Regeneration Research Group, Department of Biology, KU Leuven, Leuven Brain Institute, 3000 Leuven, Belgium; marie.claes@kuleuven.be (M.C.); emiel_geeraerts@hotmail.com (E.G.); stephanie.mentens@kuleuven.be (S.M.); 2 VIB Nucleomics Core, 3000 Leuven, Belgium; stephane.plaisance@vib.be; 3 Cellular Communication and Neurodegeneration Research Group, Department of Biology, KU Leuven, Leuven Brain Institute, 3000 Leuven, Belgium; lies.degroef@kuleuven.be; 4 Neuroplasticity and Neuroproteomics Research Group, Department of Biology, KU Leuven, Leuven Brain Institute, 3000 Leuven, Belgium; lut.arckens@kuleuven.be; 5 Neurobiology and Gene Therapy Research Group, Department of Neurosciences, KU Leuven, Leuven Brain Institute, 3000 Leuven, Belgium; chris.vandenhaute@kuleuven.be; 6 KU Leuven Viral Vector Core, 3000 Leuven, Belgium

**Keywords:** chemogenetics, DREADDs, glaucoma, retinal ganglion cells, superior colliculus, postsynaptic target area, neuroprotection, RNA sequencing, FACS, neuromodulation

## Abstract

One important facet of glaucoma pathophysiology is axonal damage, which ultimately disrupts the connection between the retina and its postsynaptic brain targets. The concurrent loss of retrograde support interferes with the functionality and survival of the retinal ganglion cells (RGCs). Previous research has shown that stimulation of neuronal activity in a primary retinal target area—i.e., the superior colliculus—promotes RGC survival in an acute mouse model of glaucoma. To build further on this observation, we applied repeated chemogenetics in the superior colliculus of a more chronic murine glaucoma model—i.e., the microbead occlusion model—and performed bulk RNA sequencing on collicular lysates and isolated RGCs. Our study revealed that chronic target stimulation upon glaucomatous injury phenocopies the a priori expected molecular response: growth factors were pinpointed as essential transcriptional regulators both in the locally stimulated tissue and in distant, unstimulated RGCs. Strikingly, and although the RGC transcriptome revealed a partial reversal of the glaucomatous signature and an enrichment of pro-survival signaling pathways, functional rescue of injured RGCs was not achieved. By postulating various explanations for the lack of RGC neuroprotection, we aim to warrant researchers and drug developers for the complexity of chronic neuromodulation and growth factor signaling.

## 1. Introduction

Within the retinofugal system, the axons of the retinal ganglion cells (RGCs) bundle together in the optic nerve, physically connecting the eye to the brain. Disruption of this linkage, as observed in optic neuropathies such as glaucoma, disconnect the RGCs from their target neurons. Not only in the visual system, but throughout the entire central nervous system, target areas are involved in safeguarding the homeostasis of innervating neurons via the production and release of growth factors [1]. In glaucoma, the so-called “neurotrophic factor deprivation theory” builds on this principle. This theory postulates that hindered retrograde axonal transport—as observed in the pathophysiology of glaucoma—causes a deficiency of target-derived neurotrophic factors in the retina, thereby rendering the RGCs prone for degeneration [2,3,4,5,6]. The precise content of the brain-to-eye axonal cargo is largely unidentified, though it is assumed that members of the neurotrophic factor family are part of it, including brain derived neurotrophic factor (BDNF) and nerve growth factor (NGF). Of note, there is also a local, retinal supply of neurotrophic factors, yet downstream intracellular signaling upon local or target-derived neurotrophic factor presentation is shown to differ. For example, local and target-derived BDNF has opposing effects on RGC dendritic arborization during development in tadpoles [7] and on extracellular signal-regulated kinase (ERK) signaling in adult rat RGCs [8]. Although much remains to be discovered regarding the differences between local and target-derived trophic support, these observations could indicate that RGCs require both supporting systems to preserve their full functionality. The current hypothesis is that target-derived factors must assist local neurotrophic support for sustained RGCs survival [9]. Indeed, in comparison to BDNF supplementation to the eye, applying BDNF to both the eye and brain enhanced and prolonged RGC survival in cats subjected to optic nerve injury [10].

**Figure 1:**
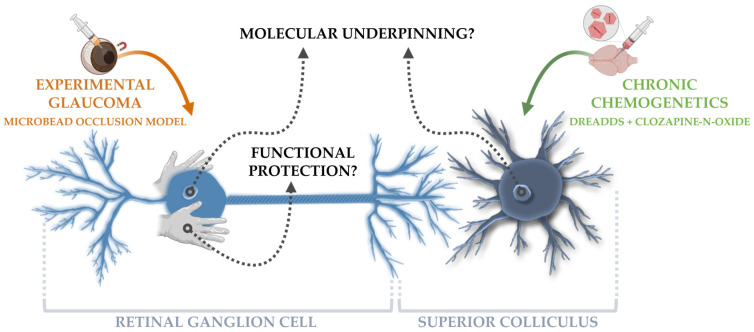
Brief overview of the study design. To explore the role of chronic target activation on the health of retinal ganglion cells (RGCs) upon glaucomatous injury, the microbead occlusion model was used to induce a glaucomatous-like pathology in the murine eye and the DREADD toolbox was applied in the superior colliculus as a chronic chemogenetic neuromodulation tool. Next, the molecular underpinning of these techniques was evaluated via bulk RNA sequencing on collicular lysates and isolated RGCs, whilst probing the functionality of injured RGCs.

Given the established link between neuronal activation and the release/transport of neurotrophic factors [11,12,13], we previously studied the effect of repeated optogenetic stimulation of postsynaptic target neurons in an acute glaucoma model. RGC neuroprotection was achieved by solely interfering with neuronal activity in one of the primary murine retinal target areas, i.e., the superior colliculus [14]. To increase the translational value of our findings, we aimed to validate our results in a more chronic experimental toolbox: optogenetics was replaced by chemogenetics and the acute and rather drastic glaucoma model by a chronic and milder one. Next, to further unfold how chemogenetic activation of postsynaptic target neurons impacts RGC survival in the glaucomatous retina, bulk RNA sequencing was used to explore the underlying transcriptional programs. The molecular responses of the superior colliculus cells to repeated collicular neuronal stimulation, as well of the unstimulated RGCs, were mapped to gain new insights into how retinal neurons rely on their target connections when embedded in an impaired circuitry (Figure 1).

In this study, the leading chemogenetic platform—i.e., Designer Receptors Exclusively Activated by Designer Drugs (DREADD) —was used [15,16]. Over the past years, DREADDs have quickly gained popularity as a plug-and-play tool in diverse research studies, especially in the field of chronic neuromodulation. Unfortunately, despite its rapid implementation, the fundamental understanding of the molecular mechanisms underlying chronic DREADD activation is still limited [17]. As such, our transcriptomic study provides one of the first reports about the molecular signature of chronic DREADD activation in rodents and could be of great interest to scientists implementing the DREADD toolbox into their research, and to the chemogenetic field in general.

## 2. Materials and Methods

### 2.1. Experimental Animals

Experiments started when mice reached the age of five weeks. Mice of either sex (2–4 animals/cage) were randomly assigned to each experimental group. All animals were bred in-house under standard laboratory conditions (14 h/10 h light/dark cycle with ad libitum access to food and water). All experimental procedures were approved by the Institutional Ethical Committee of KU Leuven (P007/2018).

To optimize the DREADD toolbox (Section 2.3), a cohort of 55 C57BL/6J mice (https://www.jax.org/strain/000664, accessed on 23 May 2022) was used (*n* = 4–6 per experimental group). For the bulk RNA study, Vglut2-ires-Cre × Thy1-STOP-YFP mice were used (*n* = 10–12 per experimental group). Vglut2-ires-Cre (https://www.jax.org/strain/016963, last accessed on 23 May 2022) and Thy1-STOP-YFP (https://www.jax.org/strain/005630, last accessed on 23 May 2022) mice were crossed to render a transgenic line that uniquely labels retinal ganglion cells (RGCs), all as described by Tran et al. [18]. Retinas of C57BL/6J and Thy1-YFP-16 (https://www.jax.org/strain/003709, last accessed on 23 May 2022) mice were employed as negative and single-color controls, respectively, for fluorescence-activated cell sorting (FACS). The in vivo measurements—i.e., intraocular pressure (IOP), anterior chamber depth or positive scotopic threshold recordings (pSTR)—were performed on the cohort of mice from the bulk RNA sequencing study and an additional cohort of Vglut2-ires-Cre × Thy1-STOP-YFP mice, rendering a sample size of 20–24 mice per experimental group.

### 2.2. Stereotactic Viral Vector Injections

Viral vectors were produced and purified by the Leuven Viral Vector Core (https://gbiomed.kuleuven.be/english/corefacilities/LVVC/, accessed on 23 May 2022), as previously described [19,20]. Briefly, AAV2/7 viral vectors encoding an hM3D(Gq)-mCherry construct or mCherry transgene—referred to as “DREADD” or “null” vectors, respectively—under the control of a CaMKIIα promoter were produced. The hM3D(Gq) sequence was based on the one published by the Roth lab, available via https://www.addgene.org/44361/ (accessed on 23 May 2022) [21]. Both vectors were used at a dilution of 1:8 in phosphate-buffered saline (PBS), with final titers of 7.91 × 10^11^ and 2.60 × 10^12^ genomic copies/mL for the DREADD and null vector, respectively, as determined via real time qPCR.

**Figure 2:**
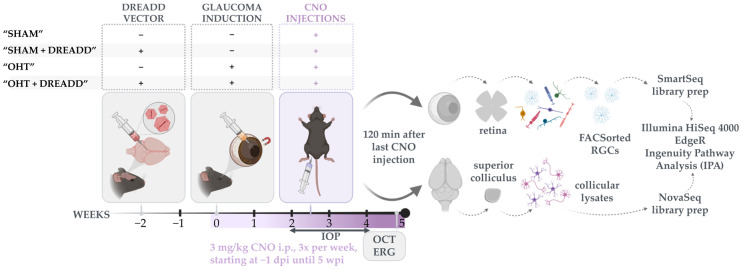
Schematic diagram of the experimental design. Two weeks after DREADD or null vector injections in the superior colliculus, ocular hypertension (OHT) was induced via intracameral injection of magnetic microbeads versus saline for SHAM controls. All mice received clozapine-N-oxide (CNO) injections (i.p., 3 mg/kg, 3 days/week), starting 1 day before glaucoma induction until five weeks later. As depicted, four experimental groups were included in this study: “SHAM”, “OHT”, “SHAM + DREADD” and “OHT + DREADD”. Assessed read-outs were longitudinal intraocular pressure measurements (IOP) and several endpoint (five weeks post injury) measurements: optical coherence tomography (OCT), electroretinography (ERG), and bulk RNA sequencing. For the bulk RNA sequencing study, both collicular lysates, as well as isolated RGCs via fluorescence-activated cell sorting (FACS) were collected. Downstream analysis was performed with EdgeR and Ingenuity Pathway Analysis (IPA).

Stereotactic viral vector injections in the superior colliculus were performed as previously described [14], with minor adaptations. Briefly, 5-week-old mice were administered general and local anesthetics 15 min before surgery. General anesthesia was induced via an intraperitoneal (i.p.). injection of medetomidine and ketamine (1 mg/kg bodyweight, Domitor, Pfizer, New York City, NY, USA and 75 mg/kg, Anesketin, Eurovet, Bladel, The Netherlands), whereas local anesthesia was administered subcutaneously (s.c.) at the injection site (5 mg/kg bupivacaine, Marcaine, Aspen, Gorinchem, The Netherlands). To avoid corneal dehydration during surgery, ophthalmologic ointment (DuraTears, Alcon, Fort Worth, TX, USA) was applied on both eyes. A Burr hole was drilled above the left superior colliculus (−0.5 to −0.7 mm relative to the Lambda coordinate). A total volume of 200 nl DREADD or null vector was injected (4 injections of 50 nl over a span of 2 min) at a depth of −1.3 mm relative to the pia mater, via a pulled-glass capillary (30 µm tip diameter) attached to a nanoliter injection (Nanoject II microinjector, Drummond Scientific, Broomall, PA, USA). After surgery, general anesthesia was reversed with 1 mg/kg atimapezol (Antisedan, Pfizer). Before surgery and twice daily for 48 h postoperative, mice received s.c. injections of the analgesic buprenorphine (1 mg/kg, Vetergesic, Ceva, Libourne, France) for pain relief. Two weeks after viral vector injections, mice were weighed and injected intraperitoneally with clozapine-N-oxide (3 mg/kg in 0.9% NaCl, CNO, Tocris, Bristol, UK) for five consecutive weeks. CNO injections were administered three times per week—Monday, Wednesday and Friday morning between 9 and 11 AM—starting one day before glaucoma induction (Cfr. Figure 2). The last CNO injection was given 2 h before euthanasia. To account for any off-target effects of CNO or clozapine, all mice received CNO injections, regardless of the experimental group [17].

### 2.3. DREADD Optimization

#### 2.3.1. Exclusion of Viral Vector Transport to the Retina

To exclude retinal transduction, the DREADD viral vector was injected in the superior colliculus at different dilutions (1:2, 1:4 and 1:8 in PBS). Two weeks after stereotactic injection, mice were euthanized with an overdose of sodium pentobarbital (60 mg/kg, Dolethal, Vetoquinol, Aartselaar, Belgium) and transcardially perfused with 0.9% saline and 4% paraformaldehyde (PFA) in PBS. Retinas were dissected, flatmounted, and double stained with anti-Brn3a (1:750, Santa-Cruz Biotechnologies, Dallas, TX, USA) and anti-RFP (1:5000, Rockland, Philadelphia, PA, USA), as previously described [14].

#### 2.3.2. Neuronal Activation and Receptor Desensitization upon Chronic DREADD Activation

To study neuronal activation upon chronic DREADD stimulation, mice were injected intraperitoneally with saline (“acute” group) or CNO (1 or 3 mg/kg, “chronic” group) for 3 consecutive weeks, two weeks after stereotactic injection of the DREADD or null vector. Mice were dark adapted the night before the final CNO injection. Two hours post injection, mice were euthanized in the dark and perfused brains were dissected, post-fixed overnight in 4% PFA and embedded in 4% agarose (Ultrapure Agarose, Invitrogen, Waltham, MA, USA in PBS) and 50 µm-thick coronal vibratome (Microm HM 650 V, Thermo Fisher Scientific, Waltham, MA, USA) sections were cut at the level of the superior colliculus (from Bregma −4.90 to −2.80 mm). A series of 8 slices, 300 µm spaced, ranging from the caudal to the rostral part of the superior colliculus, was stained with anti-RFP (1:2000, Chromotek, Planegg, Germany) and 4′,6′-diamidino-2-phenylindole (DAPI, 1:1000, Sigma-Aldrich, Saint Louis, MO, USA) to study viral vector transduction. Sections located around Bregma −3.80 mm were double stained for c-Fos (1:40,000, in-house produced c-Fos antibody [22]). Thereto, free-floating sections were permeabilized, submerged in a solution of 1% hydrogen peroxide (Chem-lab, Zedelgem, Belgium), rinsed three times and incubated overnight with rat anti-RFP and rabbit anti-c-Fos, diluted in PBS + 0.3% Triton X-100 (Thermo Fisher Scientific). After rinsing (3× with PBS + 0.3% Triton X-100) sections were incubated with biotinylated donkey anti-rabbit (1:300, Jackson ImmunoResearch, West Grove, PA, USA) and donkey anti-rat Alexa 594 (1:200,Invitrogen) in Tris-NaCl-blocking (TNB) buffer for 45 min. After rinsing, signal amplification steps were applied using the TSA FITC kit (PerkinElmer, Waltham, MA, USA), according to the manufacturer’s instructions. After washing in PBS (3×), sections were submerged in DAPI for nuclear counterstaining (30 min) and mounted on gelatin coated glass slides with the anti-fading medium Mowiol (10%, Sigma-Aldrich). Mosaic pictures were acquired with a wide-field epifluorescence microscope (Leica DM6, Wetzlar, Germany) using a 10× objective. C-Fos expression was quantified using an in-house Fiji script (ImageJ, v1.53o, Bethesda, MD, USA) [23]. Briefly, the transduced area (RFP^+^) was manually outlined and the c-Fos-immunopositive (c-Fos^+^) nuclei were counted within the RFP^+^ area, using the auto threshold Otsu and the Analyze Particles tool.

### 2.4. Experimental Glaucoma Model and In Vivo Read-outs to Probe Glaucoma Induction and Neuroprotection

The microbead occlusion model was used as experimental glaucoma model and induced as previously described [24]. Briefly, magnetic microbeads were injected in the anterior chamber of the dilated (Mydriacyl, Novartis Pharma, Basel, Switzerland) right eye under general anesthesia (4% isoflurane for induction, 1.5% isoflurane for maintenance in 96% O2, Iso-Vet 1000 mg/g, Dechra, Northwich, UK). The microbeads were manually re-positioned towards the iridocorneal angle via a handheld magnet. Sham control mice received an intracameral injection with an equivalent volume of vehicle (balanced salt solution, BSS, Alcon). Animals were euthanized at five weeks post injury.

The IOP was assessed by the same experienced operator in awake mice with a calibrated rebound tonometer (Tono-lab, iCare, Vantaa, Finland). The IOP was measured weekly between 9 and 11 A.M.: at −1 and 1 week post injury for habituation and at 2, 3 and 4 weeks post injury as read-out. Given the intra- and inter-animal variability of this read-out, a cumulative IOP was calculated and defined as the area under the curve of IOP over this three-week timeframe. Optical coherence tomography (OCT, Envisu R2210, Bioptigen, Morrisville, NC, USA) and electroretinography (Celeris, Diagnosys, Lowell, MA, USA) protocols were performed one day (non-CNO administration day) before euthanasia under general anesthesia with a ketamine/medetomidine mixture (Cfr. Section 2.2). Mice were dark adapted overnight, after which the eyes were dilated (0.5% tropicamide, Tropicol, Théa, Wetteren, Belgium and 15% phenylephrine hydrochloride, Phenylephrine, Théa) and pSTR responses were recorded (50 white light flashes, 0.0001 cd·s/m²). Immediately after the pSTR protocol, the anterior segment of the eye was imaged via OCT to analyze the anterior chamber depth. Anterior chamber depth and pSTR amplitudes/latencies were calculated as previously described [24]. The pupil diameter was measured on the OCT images, and mice without proper pupil dilation (< 1 mm diameter, 11 out of 89 mice) were excluded from the pSTR results. For the bulk RNA sequencing study, all mice were included, even those showing a minor IOP elevation or anterior chamber depth increase.

### 2.5. Bulk RNA Sequencing on Sorted RGCs and Collicular Lysates

Vglut2-ires-Cre × Thy1-STOP-YFP were sacrificed by cervical dislocation 2 h after the last CNO injection, after which the eyes and brains were harvested via fast dissection. The superior colliculus was dissected on ice with a prechilled scalpel, whereafter the tissues were snap frozen in liquid nitrogen and stored at −80 °C until RNA isolation. After enucleation, the retinas were dissected in preheated (37 °C) Ames’ medium (Sigma-Aldrich) supplemented with 2% heat inactivated fetal bovine serum (FBS, Gibco, Waltham, MA, USA). Retinal dissociation into a single-cell suspension was based on the protocols described by Park et al. [25] and Tran et al. [18] and performed in a biosafety cabinet. All solutions were HEPES buffered (25 mM, pH 7.4, Invitrogen). The retinas were enzymatically dissociated in papain (10 U/mL, Worthington, Columbus, OH, USA) for 30 min at 37 °C, followed by inactivation with ovomucoid (0,3% and 0,5% in bovine serum albumin, Worthington). After papain incubation, samples were kept at 4 °C and retinas were dissociated by gentle trituration in Ames’ medium supplemented with 1 mM EDTA (Acros Organics, Geel, Belgium), 2% FBS and DNAse (100 U/mL, Worthington). Next, cells were filtered through a 35 µm cell strainer (Falcon®, Corning Life Science, Corning, NY, USA) and incubated with the live-dead marker 7-AAD (BD Pharmingen, San Diego, CA, USA) for 10 min. Fluorescence-activated cell sorting (FACS, Sony SH8000, Tokyo, Japan, 100 µm nozzle size, semi-purity sort mode, sample pressure 6) was used to isolate living RGCs (defined as 7-AAD^−^/YFP^+^ cells). RGCs were sorted in low adhesion Eppendorf tubes containing RNA lysis buffer (Zymo Research, Irvine, CA, USA) with RNAse inhibitor (80 U/mL, Invitrogen) and stored at −80 °C until RNA isolation. Voltages and gates were set using wild-type C57BL/6J (negative control and single-color control for 7-AAD) and Thy1-YFP-16 (single-color control for YFP) mice. On average ± 25K living RGCs were sorted per retina (±70% sort efficiency).

### 2.6. RNA Sequencing

RNA was extracted using the DirectZol (Zymo Research, Irvine, CA, USA) for RGC samples and using TRI reagent (Life Technologies, Carlsbad, CA, USA) and the RNeasy Micro kit (Qiagen, Hilden, Germany) for collicular samples, according to the manufacturer’s instructions. RNA quality was assessed using a Bioanalyzer (Agilent Technologies, Santa Clara, CA, USA) and only samples with RNA integrity number (RIN) higher than 8 were included in the study. Samples were submitted to the KU Leuven Genomics core for bulk RNA sequencing (https://www.genomicscore.be/, last accessed on 23 May 2022). Per experimental group, 10–11 biological replicates were used and one sample equaled RGCs isolated from one retina or a lysate from one superior colliculus. Libraries were prepared using the Smart-Seq2 (RGC samples, Nextera XT DNA, Illumina, San Diego, CA, USA) or the QuantSeq 3’ mRNA (collicular samples, Lexogen, Vienna, Austria) protocols and sequenced on an Illumina HiSeq 4000 device (Illumina) to produce 50 bp single-end reads, obtaining an average of 16.9 (RGC samples) and 6.6 (collicular samples) million reads per sample, before alignment. Quality control of raw reads was performed with FastQC v0.11.7. [26] and adapters were filtered with ea-utils fastq-mcf v1.05 [27]. Splice-aware alignment was performed with HiSat2 against the Mus musculus reference genome mm10, using the default parameters. Reads mapping to multiple loci in the reference genome were discarded. Resulting BAM alignment files were handled with Samtools v1.5. [28]. Quantification of reads per gene was accomplished with HT-seq Count v2.7.14. [29,30]. Differential gene expression analysis was performed with the R/Bioconductor EdgeR package (v3.14.) [31,32] in R studio (v2021.09.1, build 372). Non-expressed genes were filtered out by requiring that more than 5 reads should have been detected in at least 2 samples.

Gene expression analysis, based on the log fold change expression, was performed with Ingenuity Pathway Analysis (IPA, Qiagen, v70750971, build ing_flintstone) [33]. The IPA input list was extracted from R without any prior cut-offs, thus including all fold-change and false discovery rate (FDR) metrics, and can be found in Supplementary File S1. The FDR cut-off of the IPA analysis was set at ≤ 0.1, whereas built-in scoring algorithms of IPA were used to rank the results of the different IPA analyses. Differentially expressed genes (DEGs) were identified using following criteria: FDR ≤ 0.1 and |log fold change| ≥ 1.3 for the RGC samples and ≥0.5 for the collicular samples. To unravel the biological mechanisms underlying our gene expression data, altered signaling pathways were pinpointed via IPA Canonical Pathway analysis (FDR ≤ 0.1, −log(*p*-value) ≥ 1.3). Overarching categories that were studied included Apoptosis; Cell Cycle Regulation; Cellular Growth; Proliferation and Development; Cellular Immune Response; Cellular Stress and Injury; Cytokine Signaling; Growth Factor Signaling; Intracellular and Second Messenger Signaling; Neurotransmitters and Other Nervous System Signaling; Organismal Growth and Development; and Transcriptional Regulation. Pathway activation or inhibition was either predicted based on the built-in z-score of IPA or via studying the visual diagram of the pathway in IPA. This diagram maps the structure of the pathway and overlays the data molecules, including information regarding the role of the affected molecules—i.e., activator or inhibitor—together with the predicted state—i.e., up- or downregulated—and the final direction of the interaction—i.e., activating or inhibiting the pathway. This way, an estimation was made on overall pathway activation or inhibition, termed “user-defined”. Next, upstream transcriptional regulators that are most likely responsible for the observed gene expression changes in our datasets were assessed via the “Regulator Analysis” function in IPA. Upstream regulators can include cytokines, enzymes, growth factors, ion channels, kinases, microRNAs, molecular groups, peptidases, phosphatases, receptors, transcription/translation/transmembrane regulators, and transporters. Of note, these upstream regulators can be DEGs, albeit this is not a prerequisite to be predicted as upstream regulator. Lists with the IPA results of the most important comparisons of this study—i.e., “OHT” vs. “SHAM” in the RGCs and “OHT + DREADD” vs. “OHT” in the RGCs and colliculus—are listed in Supplementary Files S2–S4, which include the DEGs, Canonical Pathway - and Upstream Regulator analyses of each comparison.

### 2.7. Statistics

Graphs and statistical tests (except for the IPA analysis) were extracted from Prism (GraphPad, San Diego, CA, USA, v9.3.1). Details of the statistical tests are mentioned in the figure legends. Effect sizes (Hedges’ g) were calculated with DABEST [34]. Statistical significance was set as *p* ≤ 0.05 for all analyses and data are presented as mean ± SEM.

## 3. Results

We previously showed that repeated optogenetic stimulation of the superior colliculus was able to promote RGC survival in a murine glaucoma model [14]. To increase translation value, we aimed to verify whether a more chronic approach could also protect the RGCs from ocular hypertension (OHT). Hence, optogenetics was replaced by the chemogenetic DREADD toolbox and the mild microbead occlusion model was employed. To shine light on the molecular mechanisms behind chronic DREADD neuromodulation in the superior colliculus and on the extent to which this strategy might be powerful in relation to boosting the natural mechanisms of neuroprotection upon glaucomatous injury, sorted RGCs and collicular lysates were subjected to bulk RNA sequencing. An overview of the experimental design in shown in Figure 2. Briefly, a glaucomatous-like pathology was experimentally induced via microbead occlusion of the anterior chamber. Chronically enhanced neuronal activity in the superior colliculus was achieved via vector-mediated DREADD (hM3D(Gq)) delivery and repeated CNO injections, that started one day before glaucoma induction until five weeks later. Control groups included mice injected with null vector and/or sterile saline instead of magnetic microbeads. As such, four experimental groups were included in this study, assigned as “SHAM”, “OHT”, “SHAM + DREADD” and “OHT + DREADD” (Figure 2). Glaucomatous induction and progression were assessed via different in vivo readouts, including IOP, anterior chamber depth and pSTR measurements.

**Figure 3:**
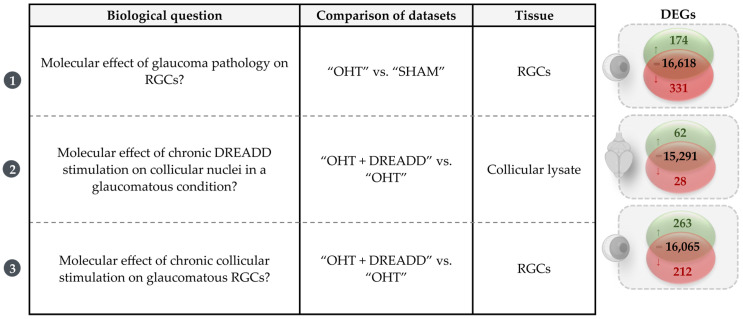
Overview of the biological questions uncovered in this study and the detected differentially expressed genes (DEGs) of these dataset comparisons. DEGs were identified based on the amplitude and statistical significance of the log fold change expression level (FDR < 0.1 and |log fold change| > 1.3 for the RGC samples vs. > 0.5 for the collicular samples). Up- and downregulated DEGs are shown in green and red, respectively, together with the unaltered genes in black.

We aimed to uncover the molecular signature of (1) glaucomatous RGCs, of (2) collicular cells upon chronic collicular stimulation in a glaucoma model, and of (3) glaucomatous RGCs upon chronic collicular stimulation (Figure 3). Bulk RNA sequencing and downstream analysis rendered 505 DEGs for the “OHT” vs. “SHAM” comparison in the RGC samples, and 90 and 475 DEGs for the OHT + DREADD” vs. “OHT” comparisons in the collicular and RGC samples, respectively (FDR < 0.1 and |log fold change| > 1.3 for the RGC samples vs. >0.5 for the collicular samples, (Figure 3)). To interpret these gene-expression results within a biological context, Canonical Pathway and Upstream Regulator analyses were performed in IPA. The Canonical Pathway analysis maps the footprint of the datasets to established signaling pathways, whereas the Upstream Regulator analysis serves to identify master transcriptional regulators that could drive the detected gene expression changes and accompanying signaling pathways. Complete lists of identified DEGs, canonical pathways and upstream regulators for the enumerated comparisons are provided in Supplementary Files S2–S4.

### 3.1. Molecular Signature of the Microbead Occlusion Model on the Retinocollicular System

**Figure 4:**
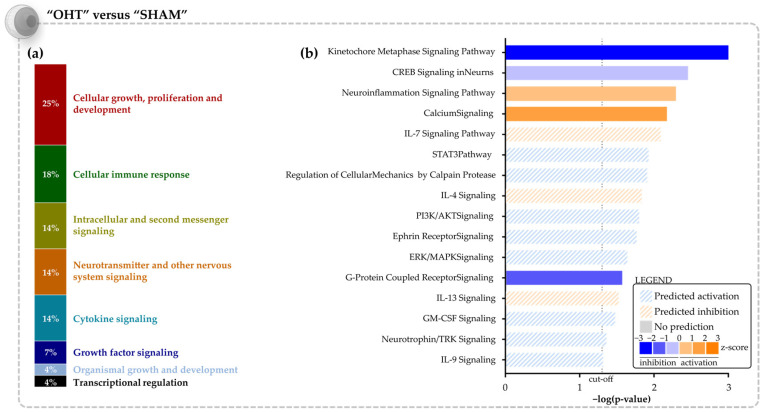
Canonical Pathway analysis in the glaucomatous RGC transcriptome (“OHT” vs. “SHAM” RGCs). (**a**) Overarching categories to which the altered canonical pathways belong. (**b**) List of altered signaling pathways relevant to the biological question (FDR ≤ 0.1, −log (*p*-value) ≥ 1.30, Fisher’s exact test). A list of all significantly altered canonical pathways is provided in Supplementary File S2. IPA predictions are algorithm- (based on z-score, filled bars) or user-defined (striped bars). Pathways that are predicted to be upregulated are shown in orange, those predicted downregulated in blue.

Five weeks after the induction of a mild glaucomatous injury via the microbead occlusion model, the transcriptome of the RGCs and collicular nuclei was studied (“OHT” vs. “SHAM”). The glaucomatous pathology did not significantly alter the transcriptome of the superior colliculus, which is therefore not included in this study. In the transcriptome of the RGCs, several DEGs were detected, some of which were found associated with an immune or inflammatory response (e.g., *Cd274, Csf2rb, Lyn, Pik3cg, Il10ra, Il12b, Ifi204* and *Sting1*) or related to a cell survival or apoptotic response (e.g., *Card11, Itga1, Acvr1c, Pik3cg*). Of note, the neurotrophic factor *Ngf* gene was significantly downregulated upon OHT. Subsequent Canonical Pathway analyses revealed significant changes in the categories Cellular Growth, Proliferation and Development, and Cellular Immune Response (Figure 4a). Various of those altered signaling pathways could be linked to neuroinflammation—e.g., Neuroinflammation Signaling, IL-7 Signaling, IL-4 Signaling and IL-13 Signaling pathways. On the contrary, several signaling pathways of the neuroprotective transcriptional network were predicted to be downregulated, including CREB Signaling in Neurons, STAT3, PI3K/AKT signaling, ERK/MAPK signaling, and Neurotrophin/TRK signaling pathways. Pathways of interest, along with their predicted activation state, are depicted in Figure 4b. Upstream Regulator analysis revealed 10 transcriptional regulators (2 activated and 8 inhibited, Supplementary File S2). Predicted inactivated regulators in glaucomatous RGCs, as compared to SHAM controls, included several interleukins (e.g., IL12B, IL4 and IL6), as well as a decreased transcriptional regulation by the pro-survival molecule STAT3 (Table 1).

### 3.2. Chronic DREADD Stimulation in the Superior Colliculus Elicits Neuronal Activation and Is Linked to Growth Factor Signaling on a Molecular Level

#### 3.2.1. Optimization of the Chronic DREADD Toolbox in the Retinocollicular System

**Figure 5:**
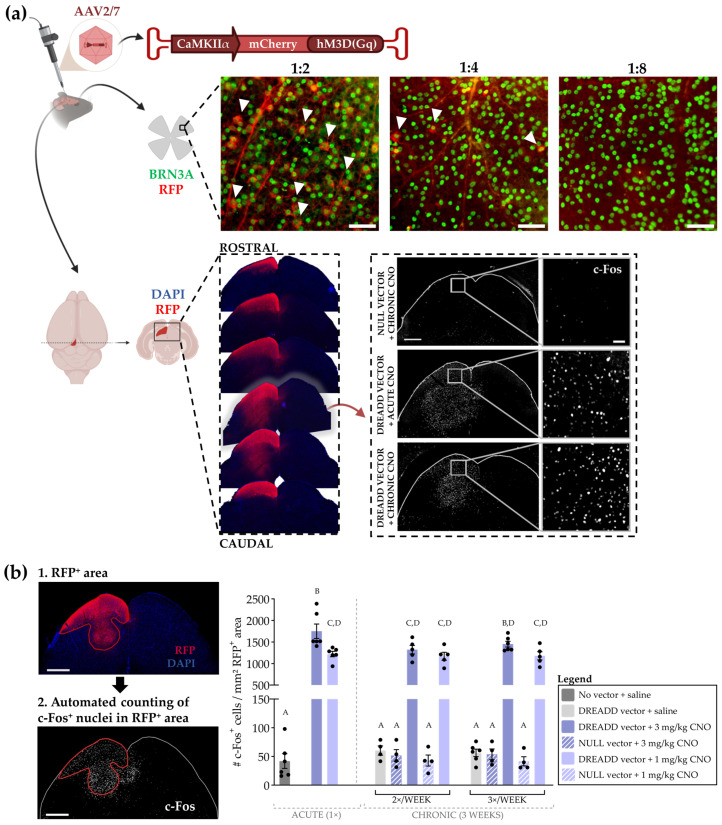
Optimization of the chronic DREADD toolbox in the retinocollicular system. (**a**) An hM3D(Gq) DREADD vector was unilaterally injected in the superior colliculus at a dilution series to exclude retrograde retinal transduction. Upon a dilution of 1:8, no transduction of RGCs was observed. For this dilution, representative images of the superior colliculus are shown, revealing a baseline c-Fos expression 2 h after CNO injection(s) upon null vector injection, versus a clear upregulation of c-Fos expression upon DREADD expression and CNO injection(s). Scale bar = 500 µm and 50 µm for magnified panels of the retina/superior colliculus. (**b**) Quantitative measurements of c-Fos^+^ density confirmed this qualitative observation: repeated CNO injections still elicit an upregulation of c-Fos expression, identical to an acute injection, independent of the CNO (3 vs. 1 mg/kg bodyweight) concentration or stimulation scheme (single injection vs. two or three times per week for three consecutive weeks). Control conditions included naive, dark-adapted mice with saline stimulation (dark grey), mice transduced with DREADD vector and saline stimulations (light grey) and mice transduced with null vector together with repeated CNO injections (striped bars), all revealing baseline neuronal activity. Uppercase letters were used to indicate statistical significance, with different letters representing significant differences (two-way ANOVA, *n*= 4–6 mice, *p* ≤ 0.05).

The chemogenetic DREADD toolbox lends itself perfectly for chronic neuromodulation, yet several hurdles related to its chronic usage have been reported in the literature [17]. The DREADD toolbox was therefore first optimized in the retinocollicular system of adult mice: a vector containing an hM3D(Gq) DREADD construct was injected into the superior colliculus and CNO was applied systemically via i.p. injections. Whilst optimizing, three parameters were evaluated: (i) retrograde transport of the viral vector, and (ii) neuronal activation by the DREADD/CNO tool and (iii) receptor desensitization upon chronic neuromodulation. To exclude the occurrence of retrograde transport of the viral vector, different dilutions of the DREADD vector (1:2, 1:4 and 1:8 in PBS) were evaluated for the presence of RGC transduction via RFP immunohistochemistry on retinal flatmounts. While minimal transduction in the RGCs was observed with higher viral vector concentrations, retrograde RGC labeling was not detected with a 1:8 vector dilution, and was thus chosen as the go-to dilution in the following experiments (Figure 5a). Secondly, DREADD desensitization upon repeated activation was assessed by comparing neuronal activity profiles of acute and chronic DREADD stimulation via the expression of the immediate early gene (IEG) c-Fos (Figure 5a). Two commonly used CNO concentrations (1 vs. 3 mg/kg bodyweight) and three different stimulation schemes (acute vs. two or three times per week for three consecutive weeks) were evaluated for the occurrence of receptor desensitization (Figure 5b). After dark adaptation and 2 h after the last CNO injection, chronic DREADD activation by CNO resulted in a clear upregulation of nuclear c-Fos expression per mm^2^ transduced area, as compared to baseline controls—i.e., naive, dark-adapted mice, mice injected with DREADD vector and saline stimulation, and mice injected with null vector and CNO stimulation—for each concentration and each injection scheme. No qualitative or quantitative differences in the neuronal activity profile were observed between acute and chronic DREADD modulation, nor between the different ligand concentrations or administration schemes (Figure 5a,b). As such, based on the c-Fos expression pattern, no indications towards hM3D(Gq) desensitization were detected. Hence, the combination of the highest concentration of CNO (i.e., 3 mg/kg) and the most frequent stimulation scheme (i.e., three times/week) were chosen to achieve maximal, and most sustained neuronal activation in further experiments.

#### 3.2.2. Molecular Signature of Chronic DREADD Stimulation in the Superior Colliculus on Collicular Cells

**Figure 6:**
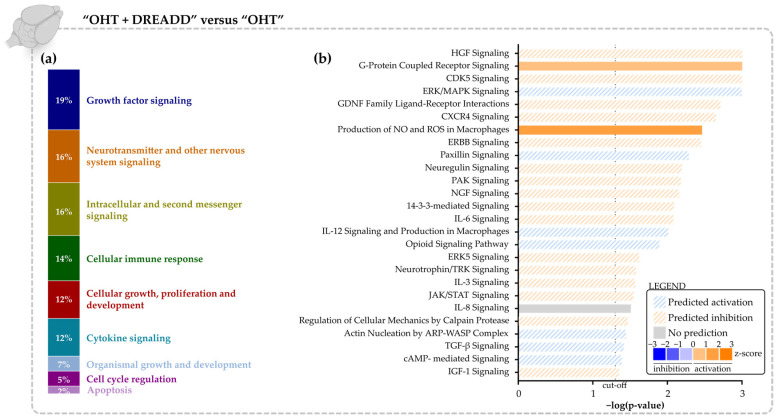
Canonical Pathway analysis in the “OHT + DREADD” vs. “OHT” dataset of the collicular transcriptome. (**a**) Overarching categories to which the altered canonical pathways belong. (**b**) List of altered signaling pathways, relevant to the biological question (FDR ≤ 0.1, −log (*p*-value) ≥ 1.30, Fisher’s exact test). A list of all significantly altered canonical pathways is provided in Supplementary File S3. IPA predictions are algorithm- (based on z-score, filled bars) or user-defined (striped bars). Pathways that are predicted to be upregulated are shown in orange, those predicted downregulated in blue. A grey bar indicates that no prediction could be made.

To decipher the molecular effect of chronic DREADD stimulation in the superior colliculus, the collicular transcriptome was studied. As the glaucoma model did not induce injury/stress signs in the superior colliculus on a transcriptomic level, and the signatures of “SHAM + DREADD” vs. “SHAM” and “OHT + DREADD” vs. “OHT” in the collicular lysates were highly similar, only the “OHT + DREADD” vs. “OHT” comparison is discussed. Chronic DREADD stimulation of neuronal activity led to a significant boost of IEGs as DEGs— e.g., *Egr1, Egr2, Nr4a1, Nr4a4, Fos, Fosb, Fosl2, Jun, Junb.* Using the Canonical Pathway Analysis in IPA, most of the altered signaling pathways (±20%) were found to belong to the Growth Factor Signaling category (Figure 6a). Growth factor signaling pathways that were predicted to be upregulated include ERBB-, GDNF-, HGF-, IGF-, NGF-, Neuregulin-, and Neurotrophin/TRK Signaling (Figure 6b). Various other enriched pathways were found to be associated with “Neurotransmitter and Other Nervous System Signaling” and “Intracellular and Second Messenger Signaling” (Figure 6a). Upstream Regulator analysis in IPA identified 72 upstream regulators (70 activated and 2 inhibited, Supplementary File S3) in the “OHT + DREADD” vs. “OHT” collicular samples. The top 20 activated transcriptional regulators included various growth factors such as EGF, IGF1, TGFB1 and neurotrophic factors BDNF, HGF and NGF (Table 2).

### 3.3. Collicular Stimulation Does Not Functionally Rescue Glaucomatous Retinal Ganglion Cells, but Induces a Partial Transcriptomic Rescue with Activation of Promising Pro-Survival Pathways

#### 3.3.1. Molecular Effects of Chronic Target Stimulation on Unstimulated, Glaucomatous Retinal Ganglion Cells

**Figure 7:**
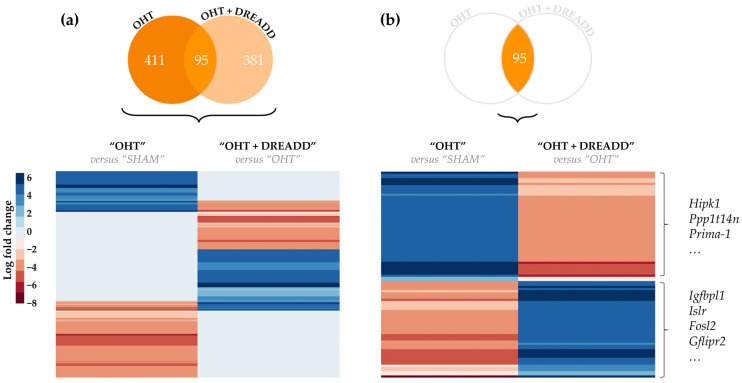
Heatmaps of the differentially expressed genes (DEGs) detected in the “OHT” vs. “SHAM” and “OHT + DREADD” vs. “OHT” comparisons of the RGC transcriptome. (**a**) Comparing the gene expression of all DEGs—i.e., unique and shared DEGs between both datasets—showed that chronic target stimulation partially reversed the disease signature. (**b**) This reversal of disease signature is emphasized when only plotting the shared DEGs. Red and blue colors denote a decreased (log fold change < −1.3) and increased (log fold change > 1.3) gene expression, respectively, whereas a white color represents genes that were not differentially expressed.

After studying the local effects of chronic target neuromodulation, we next evaluated whether the elicited changes in the superior colliculus also affected the transcriptome of downstream, unstimulated glaucomatous RGCs. Gene expression signatures—i.e., the DEGs—of “OHT” and “OHT + DREADD” RGCs were plotted (Figure 7a,b). Interestingly, the glaucomatous disease signature in the RGCs was—although only partially—reversed by chronic collicular stimulation. Interesting genes that were upregulated in glaucomatous RGCs yet downregulated in such RGCs after brain target stimulation include pro-apoptotic regulators *Hipk1, Ppp1t14n* and *Prima-1*. Conversely, *Igfbpl1, Islr*, *Fosl2* and *Gflipr2* are examples of genes that were downregulated in glaucomatous RGCs and upregulated upon target treatment, which are suggested to be involved in RGC protection and/or axonal growth. The entire list of shared DEGs between both datasets (“OHT” vs. “SHAM” and “OHT + DREADD” vs. “OHT”) can be found in Supplementary File S5.

**Figure 8:**
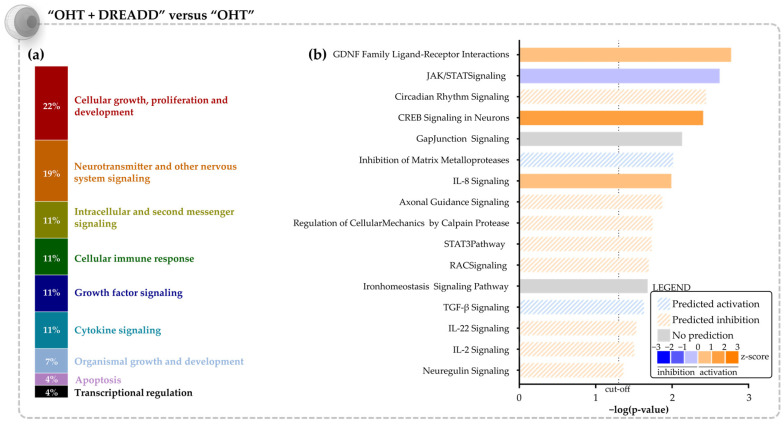
Canonical Pathway analysis in the “OHT + DREADD” vs. “OHT” dataset of the RGC transcriptome. (**a**) Overarching categories to which the altered canonical pathways belong. (**b**) List of the altered signaling pathways, relevant to the biological question (FDR ≤ 0.1, −log (*p*-value) ≥ 1.30, Fisher’s exact test). A list of all significantly altered canonical pathways is provided in Supplementary File S4. IPA predictions are algorithm- (based on z-score, filled bars) or user-defined (striped bars). Pathways that are predicted to be upregulated are shown in orange, those predicted downregulated in blue. A grey bar indicates that no prediction could be made.

Next, the transcriptome of unstimulated glaucomatous RGCs upon chronic collicular stimulation was studied in more detail (“OHT + DREADD” vs. “OHT”). The DEGs in the RGC transcriptome were found to be linked to the regulation of apoptosis (e.g., *Bard1, Stat5, S100a4, Egfr1, Nr4a1, Bmp2* and *Ckn1a*) and inflammation (*Cebpb, Il22, Il17a* and *Il20a*) (Supplementary File S4). Moreover, an IEG response was also detected in these RGCs (e.g., *Egr1, Egr2, Nr4a1, Fos, Fosl2)*. The Canonical Pathway analysis revealed that the most significantly affected overarching categories are “Cellular growth, proliferation and development”, and “Neurotransmitter and other nervous system signaling” (Figure 8a). Reflecting on our hypothesis—i.e., target stimulation aids injured RGCs, potentially via retrograde transport of neurotrophic factors—several of the detected pathways could be linked to RGC survival and/or axonal outgrowth. Examples include the GDNF Family Ligand-Receptor Interactions, CREB Signaling in Neurons, IL-8 Signaling, STAT3, RAC Signaling, TGF-β Signaling, IL-2 Signaling and Neuregulin Signaling pathways (Figure 8b). Notably, CREB Signaling in Neurons and STAT3 pathways were altered in the opposite direction as compared to the “OHT” vs. “SHAM” transcriptome of the RGCs, i.e., decreased upon glaucomatous injury, yet activated upon application of chronic DREADD stimulation in the superior colliculus. In total, 24 upstream regulators (21 activated and 3 inhibited, Supplementary File S4) were identified by IPA. Predicted activated upstream regulators comprised various growth factors—e.g., AGT, TGFB3 and FGF21, including neurotrophic factors BDNF, HGF and NGF (Table 3). Growth factors that were indicated as important upstream regulators were, however, not detected as DEGs. Neurotrophic receptor tyrosine kinase 1 (NTRK1)—which is regulated by neurotrophins NGF, NTF3 and BDNF—was also appointed as an activated transcriptional regulator.

#### 3.3.2. Chronic Collicular Stimulation Does Not Alleviate RGC Injury in Glaucomatous Mice

**Figure 9:**
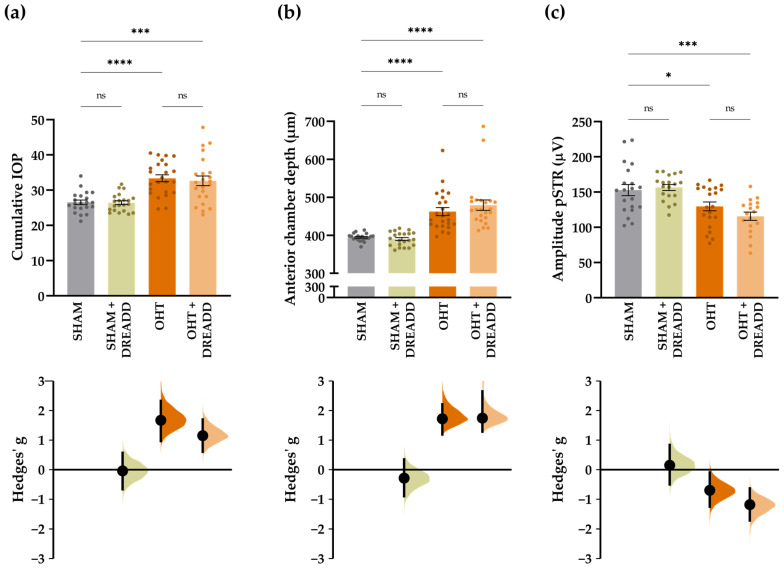
One-way ANOVA and estimation statistics (Hedges’ g) on different read-outs to probe glaucoma induction and progression (*n* = 18–24 mice). (**a**) Cumulative IOP, defined as the area under the curve of IOP measured at 2, 3 and 4 weeks post injury, was elevated upon OHT induction—i.e., in “OHT” and “OHT + DREADD mice”—and unaltered in “SHAM + DREADD” mice as compared with “SHAM” controls. (**b**) Similarly, anterior chamber depth was elevated five weeks after OHT induction, yet not upon DREADD stimulation alone as compared with “SHAM” controls. (**c**) “SHAM + DREADD” mice showed a similar pSTR response as “SHAM” mice, whereas this response was significantly lower in “OHT” and “OHT + DREADD” mice as compared with “SHAM” mice. The pSTR response was identical in “OHT” and “OHT + DREADD” mice, revealing that DREADD stimulation of the superior colliculus did not rescue RGC functionality in mild glaucomatous mice. Mice with a pupil diameter < 1 mm were excluded. Key: ns = non-significant, * *p* ≤ 0.05, *** *p* ≤ 0.001 and **** *p* ≤ 0.0001. Mean values and estimation statistics are listed in Supplementary File S6, Tables S1 and S2.

To explore the effect of chronic target stimulation on RGC functionality in our mild glaucomatous pathology, three in vivo read-outs were assessed: IOP, anterior chamber depth and pSTR measurements (Figure 9a–c). All measurements were performed on each of the four experimental groups—i.e., “SHAM”, “OHT”, “SHAM + DREADD” and “OHT + DREADD”. To start, DREADD stimulation without the induction of OHT (“SHAM + DREADD”) had no effect on any measured parameter: “SHAM + DREADD” mice show no rise in IOP, no increase in anterior chamber depth and no changes in pSTR amplitude as compared with “SHAM” mice. Induction of glaucomatous pathology, however, significantly raised the IOP (+26.29 ± 3.45%) and anterior chamber depth (+17.20 ± 2.70%) in “OHT” mice as compared with “SHAM” controls (Figure 9a,b). DREADD stimulation in OHT mice (“OHT + DREADD”) did not affect these inclines: +24.62 ± 4.77% and +21.48 ± 3.45% for the IOP and anterior chamber depth measurements, respectively (Figure 9a,b). As previously reported, the murine microbead occlusion model induces a mild pathology in which early signs of RGC injury are best evaluated via functional pSTR recordings [24]. Indeed, the amplitude of STR recordings is significantly lower in “OHT” mice (−16.40 ± 3.85%) as compared to “SHAM” mice. Unfortunately, DREADD stimulation in OHT mice did not avert this decline in RGC functionality as the pSTR response of “OHT + DREADD” mice was still significantly reduced, as compared to “SHAM” (−24.47 ± 3.74%) and “SHAM + DREADD” mice (−26.10 ± 3.50%), and no difference in pSTR response was observed between “OHT” and “OHT + DREADD” mice (Figure 9c). As such, chronic chemogenetic stimulation of the superior colliculus did not alleviate RGC damage in the microbead occlusion model.

## 4. Discussion

The overall goal of this study was two-fold; we aimed to evaluate (i) the molecular signature of the superior colliculus after artificially and chronically enhancing neuronal activation, and (ii) whether this chronic target stimulation could contribute to the health of connecting, unstimulated RGCs upon glaucomatous injury, both on a transcriptional and functional level.

### 4.1. Growth Factors Are Key Regulators of the Transcriptomic Changes upon Chronic DREADD Activation

There is a dynamic interplay between neuronal activation and neurotrophic factor signaling, which is highly evidenced for the neurotrophic factor BDNF. Regulation of BDNF is shown to be activity-dependent: upon neuronal activation, its expression [35,36,37,38] and secretion [39,40,41,42,43] is augmented, pro-BDNF is converted into mature BDNF [39] and its receptor Tropomyosin receptor kinase B (TRKB) is recruited to the plasma membrane [44,45,46]. Given this tight linkage, we hypothesized that artificially stimulating neuronal activation via DREADDs could also induce a gulf of BDNF or other neurotrophic factors. As neurotrophic factors are considered as generic mediators of neuronal homeostasis and survival, this elicited gulf could entail neuroprotective effects after glaucomatous injury.

Profound knowledge on the precise molecular underpinning of (chronic) DREADD activation is largely lacking. Few studies that have offered an unbiased interrogation of the underlying transcriptomic/proteomic profiles, as reviewed in [17], have highlighted an important role for BDNF signaling. For example, Hallock et al. [47] found an activity-dependent upregulation of several genes in the BDNF-TRKB signaling pathway after chemogenetic activation of the medial prefrontal cortex in the adult murine brain. Similarly, RNA sequencing pinpointed BDNF signaling as an important mediator of suppressing antianxiety-like behavior and neurogenesis upon chemogenetic activation of dorsal dentate gyrus neurons in adult mice [48]. Targeted studies to evaluate BDNF signaling upon chemogenetic neuronal activation also confirmed an upregulation of BDNF. Xia et al. [49] have reported that BDNF protein levels decreased upon silencing of dopaminergic neurons and that, vice versa, neuronal activation increased BDNF levels. Similarly, Blázquez et al. [50] have confirmed increased *Bdnf* mRNA levels upon activation of dorsolateral striatal neurons in mice. Lastly, Xiu et al. [51] showed beneficial effects of BDNF supplementation in mouse models of obesity and diabetes, effects that could be mimicked with chronic neuronal activation of the dorsal raphe nucleus. In our study, an important role is also assigned to growth factors in general, and neurotrophic factors in particular. Although not detected as DEGs at the sampled timepoint, various enriched signaling pathways—e.g., GDNF-, HGF-, NGF- and Neurotrophin/TRK Signaling pathways—and upstream regulators—e.g., BDNF, HGF and NGF—can be linked to neurotrophic factor signaling. Our results provide additional proof that in vivo stimulation of neuronal activity in the superior colliculus via DREADDs induces transcriptomic regulation by growth factors, even after chronic neuromodulation.

### 4.2. Transcriptomic Analysis Confirms the Pro-Survival Effects of Chronic Collicular Stimulation on Glaucomatous Retinal Ganglion Cells

Throughout the central and peripheral nervous system, neurotrophic factors are well-known for their role as long-distance, retrograde modulators [43,52,53,54,55]. In the visual system, several reports have indeed proposed that neurotrophic factors are part of the retrograde cargo that is transported towards the RGCs. Neurotrophic factors were shown to travel retrogradely towards the retina when exogenously supplied in RGC target areas in the adult rodent brain [56,57,58,59], as reviewed in [9]. Moreover, RGCs respond to BDNF administration in a postsynaptic target area of developing tadpoles, a response that could be abolished by blocking axonal transport or TRKB receptors [60]. According to the neurotrophic factor deprivation theory in glaucoma, these retrogradely transported factors are crucial for RGC survival upon injury. Indeed, Chou et al. showed that RGC functionality declines when retrograde signaling is impaired in the adult mouse visual system. The authors link this observation with a lack of target-derived factors and propose BDNF as the most likely candidate [61]. As such, neurotrophic factors, and especially BDNF, are deemed important cargo on the axonal highway throughout the nervous system, including the retinocollicular system.

Given the detected boost of growth factors upon chronic collicular stimulation (Cfr. Section 3.2) and their role as retrograde modulators of neuronal survival, we hypothesized that this boost could affect the survival of unstimulated, connecting RGCs via retrograde signaling. Indeed, on top of a local involvement of growth factors in the stimulated area, chronic target stimulation also elicited a similar response in the transcriptome of distantly located RGCs. The neurotrophic factors BDNF, HGF and NGF were predicted as activated upstream regulators in both the RGC and collicular samples upon chronic DREADD stimulation in mild glaucomatous mice. Moreover, the signaling pathway CREB Signaling in Neurons was found to be enriched, and several IEGs like *Fos* were detected as DEGs (Cfr. Section 3.3). Axonal BDNF signaling is indeed shown to exert its effects via CREB signaling [55,62,63] and early gene expression [55,63,64]. This emphasizes the occurrence of retrograde growth factor signaling upon chronic neuronal stimulation of collicular nuclei, possibly contributing to pro-survival and/or pro-regenerative effects upon glaucomatous injury.

Both BDNF and NGF are known promoters of RGC survival upon glaucomatous injury and their application as potential neuroprotective drugs in glaucoma management is well-studied [65]. BDNF has also been pushed forward as the most likely retrograde modulator, while other factors, such as hepatocyte growth factor (HGF), could also be involved. In the superior colliculus, HGF signaling is amongst the top enriched signaling pathways. Studies on HGF in literature are limited, especially in comparison to studies employing BDNF or NGF, yet its retinal expression and neuroprotective/regenerative effects after lesion have been studied in adult rodents [66,67,68]. Wong et al. compared the protective and regenerative effects of BDNF, CNTF and HGF and concluded that HGF excels in long-term neuroprotection and regeneration of RGCs upon axonal injury [68]. These studies, however, also pinpoint how the neuroprotective functions of HGF are correlated with its concentration and how relatively high levels of HGF are needed to confer neuroprotection. While HGF was not detected as a DEG in the RGC transcriptome of DREADD stimulated glaucomatous mice, our study suggests that studying HGF signaling upon glaucomatous injury might be a fruitful area for further work.

#### Growth Factor Signaling Versus Axonal Regeneration and Dendritic Remodeling

Chronic chemogenetic activation of neurons has been previously reported to stimulate axonal regeneration [69,70,71]. As such, it is possible that target stimulation also affects the axonal compartment and instigates axonal repair/regrowth. Varadarajan et al. have shown that chronic DREADD stimulation of a postsynaptic target area after a distal optic nerve crush promotes regeneration of adult RGCs, yet the authors did not report on RGC loss and concurrent rescue [72]. Also in our study, some hints towards an effect on axonal regeneration can be found. Indeed, the canonical pathway Axonal Guidance was predicted to be activated, and the pleiotropic cytokine interleukin-6 (IL-6) was detected as an important activated upstream regulator in the RGC transcriptome. IL-6 is typically associated with pro-regenerative capacities in injured RGCs [73,74,75], and it has been shown that IL-6 deficiency negatively impacts axonal transport and structure [76]. Other pathways linked with RGC regeneration upon optic nerve injury that were enriched in our study are STAT3 [77,78] and RAC [79] Signaling. Besides axonal degeneration, alterations in dendritic arbors are also denoted upon RGC damage in multiple animal models of glaucoma [18,24,80,81,82,83,84,85,86]. Interestingly, the RAC Signaling pathway has also been linked with dendritic remodeling of RGCs [87]. Similarly, CREB Signaling (Cfr. Section 4.2) has been shown to be involved in retrograde BDNF-induced dendritic arborization in cortical neurons [55].

### 4.3. Why Do These Pro-Survival Cues Not Result in Improved RGC Health?

We previously showed that repeated optogenetic stimulation attained RGC survival in an acute laser photocoagulation glaucoma model [14]. The current study reveals a boost of growth factors and the enrichment of several pro-survival and/or pro-regenerative pathways in the glaucomatous RGCs upon chronic chemogenetic activation of superior colliculus nuclei, all hinting towards a retrogradely induced protection of RGCs. Unexpectedly, our strategy did not confer (functional) neuroprotection in vivo, as measured via electroretinography—i.e., pSTR recordings. In sharp contrast, the disease signature marked by the glaucomatous injury on a transcriptomic level was partially reversed by our strategy of target stimulation. For example, several apoptosis activators—e.g., *Hipk1* [88], *Ppp1t14b* [18], and *Prima-1* [89]—were upregulated in glaucomatous RGCs, yet downregulated upon target stimulation. Conversely, genes that were downregulated in glaucomatous RGCs and upregulated upon treatment can be linked with RGC protection and/or axonal regeneration/repair, such as *Igfbpl1, Islr, Fosl2* and *Glipr2*. *Igfbpl1* is linked to IGF signaling and optic nerve protection and repair [90], whereas *Islr* is involved in NGF and GDNF signaling and axonal growth during development [91,92]. *Fosl2* contributes to the delay of RGC death upon disruption of JUN activity [93], and *Glipr2* is linked to ERK1/2 activation, which plays a key role in RGC protection after OHT [94,95]. This all highlights that the strategy of arming RGCs against glaucomatous injury by target stimulation still holds potential, yet many complex mechanisms might be at play that prevent ultimate neuroprotection. We can also not rule out that the injury introduced by our mild glaucoma model was too limited, both in terms of the extent and number of affected RGCs, to chart neuroprotective effects. However, the lack of a neuroprotective effect in our more chronic set-up could also be inherent to chronic DREADD neuromodulation (Section 4.3.1), attributable to the complexity of neurotrophic factor signaling (Section 4.3.2) or as a result of a duality in functioning of detected DEGs and transcriptional regulators (Section 4.3.3).

#### 4.3.1. Hazards of Chronic DREADD Stimulation: Receptor Desensitization and Neuroadaptive Changes

Neuromodulation via optogenetic versus DREADD stimulation is markedly different in terms of time scaling (i.e., minutes vs. hours/days, respectively), as well as the mechanism of activation (i.e., ionotropic vs. metabotropic, respectively) [15,16,96]. DREADDs are—besides their responsiveness to CNO instead of acetylcholine—identical to endogenous G protein-coupled receptors (GPCRs). Their activation sets off a chain reaction in which intracellular messengers are amplified, and numerous intracellular signaling pathways are altered. This induces a number of physiological responses, including the activation of ion channels and thus, neuronal activation [97]. As such, DREADDs provide a rather indirect way of neuronal activation, as compared with more direct optogenetic stimulation [98]. Hence, given these differences between both neuromodulation techniques, it is possible that the pro-survival effects of optogenetics cannot be extrapolated to chemogenetic stimulation.

GPCRs are also well-studied in the field of receptor desensitization, i.e., a diminished or absent receptor response upon repeated activation [99,100,101,102]. To define a suitable CNO administration scheme for chronic DREADD stimulation, neuronal activation was evaluated via the expression of the IEG c-Fos. No signs of receptor desensitization were detected, although it should be noted that c-Fos expression only gives a binary answer (yes/no) to the question—i.e., is there still neuronal activation upon repeated stimuli—and cannot be used to study firing pattern/frequency. To draw definite conclusions regarding receptor desensitization, firing rate profiles could be studied via in vivo electrophysiological recording. Up till now, an optimal CNO administration scheme that elicits continuous DREADD activation, without the occurrence of receptor desensitization remains to be studied in more detail. Examining the DEGs detected in the chronically stimulated superior colliculus reveals that most can be appointed as IEGs. This finding could indicate that our sampling was performed too early—i.e., 2 h post CNO injection—to detect ensuing gene expression changes downstream of the IEGs, yet signifies that neuronal activation was provoked upon continued DREADD stimulation. Further studies with more focus on receptor desensitization upon chronic chemogenetics are, however, recommended. One interesting approach to start with would be to compare the transcriptome of acute vs. chronically DREADD stimulated neurons.

#### 4.3.2. Complexity of Neurotrophic Factor Signaling

This study was set up with the hypothesis that artificially enhancing neuronal activity in the superior colliculus might be an unbiased approach to induce a retrograde cocktail of survival factors essential for RGC survival after injury. This way, neither the identity nor dosage of each factor has to be determined, which contrasts with a targeted approach, in which a single, predefined neurotrophic factor is often administered at high doses. Chemogenetic target stimulation might induce a more endogenous-like secretion of the currently unidentified retrograde survival cocktail. Given the high heterogeneity within the RGC population, we hypothesized that this comprehensive approach, including multiple survival factors, might be required to protect the complete array of RGCs. The current study indicates that neuromodulatory approaches are possibly not mimicking this endogenous survival cocktail, being perhaps far from a physiological response. Although used as pharmacological agents, the machinery of growth factors, and especially neurotrophic factors, is well-known to be particularly prone to receptor desensitization upon repeated presentation. Indeed, following repeated BDNF applications in retina as well as in the superior colliculus, downregulation of the TRKB receptor was observed [103,104]. Hence, researchers have been exploiting combinatorial approaches, i.e., enhancing both neurotrophic factor and TRKB signaling [105,106]. The unanticipated finding of our study—that chronic target stimulation via chemogenetics does not rescue RGC functionality after glaucomatous injury—may be attributed to the complex nature of growth factor signaling. More knowledge generating research linking the effects of neuromodulation and the complexity of neurotrophic factor machinery is thus needed.

Another plausible explanation could be that target-derived factors support RGC survival but fall short without any other coping strategy. Local neurotrophic factor supplementation in different glaucoma animal models has been extensively explored, with encouraging results as these studies have repeatedly demonstrated the undeniable neuroprotective power of neurotrophic factors. A common obstacle of those studies has been the transient character of the intervention: RGC loss was delayed, yet not prevented. This has led to the hypothesis that local neurotrophic factor availability aids the RGCs at early injury stages, but target-derived factors must come into play for long-term preservation of RGC functionality [9,10]. As we only focused on target-derived support, we cannot exclude the possibility that local intervention might be required in parallel.

#### 4.3.3. Duality in Functioning: Pro-Apoptosis or Pro-Survival?

A last explanation is the duality in the functioning of detected DEGs and predicted upstream regulators. For example, *Egr1* and *Bmp2* are upregulated in injured RGCs upon chronic target stimulation, and both are linked to the induction of apoptosis (Cfr. Section 3.3). Upregulation of *Egr1* has also been detected in the transcriptome of RGCs upon axonal injury [18,107] and *Bmp2* was upregulated in response to glaucomatous injury [108]. The expression of these genes could be part of the injury response leading to neurodegeneration, while also serving as an endogenous protective response of injured RGCs in an attempt to promote their own survival. Moreover, several of the detected upstream regulators have been shown to modulate different functions depending on the biological context, potentially rendering opposite results—i.e., cell death vs. survival. For example, TNF, HIF1A, IL3 and IL6 were predicted as important, activated upstream regulators in the glaucomatous RGCs upon chronic target stimulation, yet are known to exert bidirectional effects on neuronal survival. TNF is generally assumed to trigger neurodegeneration but it has also been reported that TNF can promote, depending on the balance of certain signaling pathways, survival signals in RGCs [109]. Similarly, HIF1A can promote RGC degeneration under hypoxia, whereas under normoxia, it provokes RGC survival [110]. IL3 and IL6 are both typical pro-inflammatory molecules and shown to be upregulated by injured RGCs following IOP elevation [74,111]. However, these cytokines can also promote RGC survival [111,112]. As the final biological function of a regulator depends on a complex interplay between diverse receptors and intracellular signaling pathways, an interplay which also depends on the site of signal initiation (dendrites vs. cell body vs. axon terminals), predicting the final outcome on RGC death or survival is highly complicated.

## 5. Conclusions

Chronic chemogenetic activation of the superior colliculus in the adult murine visual system elegantly phenocopies the a priori hypothesized activation of growth factors as upstream transcriptional regulators. This predicted boost was not confined to the stimulated area, yet also retrogradely spread to afferent, unstimulated RGCs. Despite this pro-survival boost and a partial reversal of the disease signature, the glaucomatous injury introduced by the microbead occlusion glaucoma model was not alleviated by chronic target area stimulation. This finding was unexpected as we previously showed that optogenetic target stimulation in an acute glaucoma model did promote RGC survival. The lack of neuroprotective effect upon chronic chemogenetics may be attributed to the complex intricacies of this study, including established hurdles of chronic neuromodulation, neurotrophic factor signaling, treatment strategies in glaucoma research, or a combination thereof. Some interesting future research avenues include in-depth studies of the molecular/cellular effects upon chronic neuromodulation with a focus on desensitization and other neuroadaptive changes, deciphering the complexity of continued neurotrophic factor stimulation, the evaluation of combinatorial approaches—i.e., local and target neurotrophic factor support in parallel—, and lastly, an unbiased screening of the “brain-to-eye” transportome. This could pinpoint novel, target-derived survival factors, thereby complementing the study by Schiapparelli et al., who investigated the anterograde eye-to-brain by the in vivo tagging of newly synthesized proteins in the retina and subsequent proteomic screening of the superior colliculus, the lateral geniculate nucleus and the visual cortex [113,114]. These studies will provide fruitful insights for researchers applying chronic neuromodulation via chemogenetics in their studies and for the glaucoma research field. Nevertheless, our study provides evidence to support existing hypotheses regarding neurostimulation and retrograde transport. It provides a comprehensive first glimpse on possible neurotrophic actions upon chronic stimulation of a postsynaptic target area within the glaucomatous visual system. Even though RGC rescue from glaucomatous injury was not achieved, chronic chemogenetic stimulation of the superior colliculus did partially reverse the disease signature of glaucomatous RGCs and elicited promising pro-survival signaling pathways.

## Figures and Tables

**Table 1:** Upstream regulators identified as significantly inhibited in the “OHT” RGCs as compared to “SHAM” RGCs (FDR ≤ 0.1, *p*-value of overlap ≤ 0.01, |z-score| ≥ 1.95).

Upstream Regulator	Expr Log Ratio	Molecule Type	Activation z-Score	*p*-Value of Overlap	Target Molecules in Dataset
BMP2		growth factor	−2.58	3.71 × 10^−3^	*Bmp4, Col10a1, Fgfr2, Grem1, Ifi16, Nfatc1, Pth1r, Tagln*
STAT3	0.19	transcription regulator	−2.50	2.12 × 10^−4^	*Alas2, Bcl3, Cd274, Cd46, Ciita, Ect2, Fcgr1a, Ffar2, Hla-A, Ifi16*
SMARCB1	0.02	transcription regulator	−2.24	7.12 × 10^−3^	*Bmp4, Fcgr1a, Fcgr2a, Fgfr2, Ifi16, Il12b, Sgcg*
IFNAR1	−0.23	transmembrane receptor	−2.21	8.66 × 10^−3^	*Cd274, Ciita, Hla-A, Ifi16, Il12b, Socs1*
IL12B	−4.31	cytokine	−2.20	5.00 × 10^−4^	*Il12b, Lta, Pik3ap1, S100a9, Socs1*
IL4	−0.11	cytokine	−2.12	2.29 × 10^−4^	*Bcl3, Cd274, Cd8a, Ciita, Col6a3, Csf2rb, Cybb, Ephb4, Fcgr1a, Fcgr2a*
IL6		cytokine	−2.02	2.77 × 10^−5^	*Afp, Avp, Bcl3, Cd274, Cd46, Ces1, Ciita, Csf2rb, Cybb, Fcgr1a*
BRD4		kinase	−1.98	4.40 × 10^−5^	*Bcl3, Cd274, Fosl2, Insm1, Itga1, Loxl2, Map4k1, Mmachc, Pole2*

**Table 2:** Top 20 upstream regulators identified as significantly activated in the “OHT + DREADD” superior colliculus lysates as compared to “OHT” lysates (FDR ≤ 0.1, *p*-value of overlap ≤ 0.01, |z-score| ≥ 1.95). An entire list of upstream regulators is provided in Supplementary File S3.

Upstream Regulator	Expr Log Ratio	Molecule Type	Activation z-Score	*p*-Value of Overlap	Target Molecules in Dataset
CREB1	−0.23	transcription regulator	3.91	9.08 × 10^−11^	*Apold1, Csrnp1, Dio2, Dusp1, Egr1, Egr4, Errfi1, Fos, Fosb, Gpr3, Junb, Nr4a1, Nr4a3, Per1, Sik1, Tiparp*
EGF		growth factor	3.63	4.62 × 10^−9^	*Cxcl13, Dio2, Dusp1, Dusp4, Egr1, Errfi1, Fos, Fosb, Fosl2, Junb, Nr4a1, Nr4a3, Per1, Serpina3, Sh2b3*
IL1B	0.40	cytokine	3.51	7.74 × 10^−5^	*Csrnp1, Cxcl13, Ddit4, Dusp1, Egr1, Errfi1, Fos, Fosb, Junb, Nr4a1, Nr4a3, Serpina3, Slc2a1*
Insulin		group	3.12	9.86 × 10^−8^	*Cox6a2, Ddit4, Dusp1, Dusp4, Egr1, Errfi1, Fos, Fosb, Junb, Lpin1, Nr4a1, Nr4a3, Per1, Slc2a1*
CREB		group	3.02	1.71 × 10^−9^	*Cox6a2, Dusp1, Dusp4, Egr1, Egr4, Fos, Fosb, Junb, Nr4a1, Nr4a3, Per1, Slc2a1*
GPER1	0.28	G-protein coupled receptor	2.91	6.92 × 10^−13^	*Ddit4, Dusp1, Dusp4, Egr1, Errfi1, Fos, Fosb, Rasd1, Slc2a1*
IGF1	0.16	growth factor	2.90	2.10 × 10^−4^	*Ddit4, Dusp1, Egr1, Errfi1, Fos, Fosb, Junb, Nr4a1, Slc2a1*
TGFB1	0.31	growth factor	2.88	2.81 × 10^−5^	*Cxcl13, Cyp3a7, Ddit4, Dusp1, Dusp4, Egr1, Fos, Fosb, Fosl2, Junb, Klf2, Mboat2, Nr4a1, Nr4a3, Rasl11b, Rbpms, Serpina3, Slc2a1, Srsf2*
MAPK3	−0.08	kinase	2.77	2.97 × 10^−9^	*Dusp4, Egr1, Egr4, Fos, Fosb, Fosl2, Junb, Nr4a1*
F2		peptidase	2.72	4.79 × 10^−6^	*Cxcl13, Dusp1, Egr1, Fos, Fosb, Fosl2, Junb, Nr4a3*
TNF	0.36	cytokine	2.66	9.25 × 10^−5^	*Cxcl13, Depp1, Dpys, Dusp1, Dusp4, Egr1, Fos, Fosb, Fosl2, Junb, Klf2, Nr4a1, Nr4a3, Rasd1, Rbpms, Serpina3, Slc2a1, Tst*
FOXO3	−0.13	transcription regulator	2.63	2.55 × 10^−5^	*Ddit4, Depp1, Egr1, Egr4, Fos, Fosb, Junb, Sh2b3*
RELA	0.27	transcription regulator	2.61	2.34 × 10^−4^	*Dio2, Dusp1, Egr1, Fos, Fosb, Junb, Nr4a1, Nr4a3*
GNAS		enzyme	2.60	9.85× 10^−9^	*Arl4d, Fos, Fosl2, Gdf9, Nr4a1, Nr4a3, Rasd1*
IL6		cytokine	2.60	6.17× 10^−3^	*Cxcl13, Dusp1, Egr1, Errfi1, Fos, Junb, Lyz, Serpina3*
HGF	0.01	growth factor	2.58	1.63× 10^−3^	*Dusp4, Egr1, Errfi1, Fos, Fosb, Nr4a1, Nr4a3*
Pkc(s)		group	2.57	1.07 × 10^−5^	*Dusp1, Egr1, Fos, Junb, Nr4a1, Nr4a3, Per1*
BDNF	0.03	growth factor	2.57	2.35 × 10^−6^	*Dusp1, Dusp4, Egr1, Fos, Fosb, Nr4a1, Nr4a3, Sik1, Tiparp*
CSF1	0.24	cytokine	2.54	3.36 × 10^−5^	*Dusp1, Dusp4, Egr1, Fos, Junb, Klf2, Slc2a1*
NGF	−0.27	growth factor	2.48	3.04 × 10^−7^	*Dusp1, Dusp4, Egr1, Egr4, Errfi1, Fos, Fosl2, Junb, Nr4a1*

**Table 3:** Upstream regulators identified as significantly activated in the “OHT + DREADD” RGCs as compared to “OHT” RGCs (FDR ≤ 0.1, *p*-value of overlap ≤ 0.01, |z-score| ≥ 1.95).

Upstream Regulator	Expr Log Ratio	Molecule Type	Activation z-Score	*p*-Value of Overlap	Target Molecules in Dataset
HIF1A	0.47	transcription regulator	2.88	1.12 × 10^−3^	*Bmp2, Cavin2, Ccn3, Cdkn1a, Egfr, Eng, Fos, Gck, H4c15, Il17ra*
BDNF	0.26	growth factor	2.60	8.93 × 10^−3^	*Bmp2, Cdkn1a, Cebpb, Egr1, Egr2, Fos, Limk1, Mag, Nr4a1, Ryr1*
AGT	1.70	growth factor	2.56	3.54 × 10^−4^	*Adam12, Avp, Bmp2, C5ar1, Ccn3, Cdc20, Cdkn1a, Cox7a1, Crlf1, Egfr*
TGFBR2		kinase	2.55	7.71 × 10^−3^	*Bcat1, Bcl3, C15orf48, C5ar1, Cdkn1a, Cdkn2b, Runx2, Sdc1, Sh3bp2, Stc2*
TNF		cytokine	2.53	2.76 × 10^−6^	*Arrdc3, Avp, Bcl3, Bmp2, C15orf48, C5ar1, Card6, Cbr3, Ccn3, Cd55*
FOXO3	−0.31	transcription regulator	2.52	1.05 × 10^−4^	*Bmp2, Cdc20, Cdkn1a, Cdkn2b, Dok5, Egr1, Egr2, Fos, Lcp2, Mbnl1*
IL6		cytokine	2.45	1.44 × 10^−4^	*Avp, Bcl3, Bmp2, C5ar1, Cd74, Cdc20, Cdkn1a, Cdkn2b,* *Cebpb, Crlf1*
NGF		growth factor	2.40	1.59 × 10^−3^	*Cdkn1a, Dusp8, Egfr, Egr1, Egr2, Fos, Fosl2, Hrk, Nr4a1, Tnfr, sf12a*
MAPK3	0.01	kinase	2.39	3.20 × 10^−4^	*Cdkn1a, Cdkn2b, Egr1, Egr2, Fos, Fosl2, Nr4a1*
TGFB3	0.04	growth factor	2.36	1.65 × 10^−5^	*Adam12, Ccn3, Cdkn1a, Cdkn2b, Eng, Fos, Fosl2, Htra1, Sox9*
CSF2		cytokine	2.34	3.46 × 10^−5^	*Anln, Bcl3, C5ar1, Card6, Cd74, Cdc20, Cdkn1a, Cdkn2b,* *Dscc1, Egr1*
CHUK	−0.16	kinase	2.21	6.41 × 10^−5^	*Bmp2, Ccn3, Cdkn1a, Cebpb, Crlf1, Fos, Hla-A, Il22, Mt1, Mt2*
Ngf		group	2.21	1.44 × 10^−3^	*Cdkn1a, Egr1, Fos, Hrk, Nr4a1*
FGF21		growth factor	2.21	1.09 × 10^−3^	*Avp, Cox7a1, Cpt1a, Egr1, Fos*
EP300	1.00	transcription regulator	2.20	1.26 × 10^−4^	*Adam12, Card6, Cdkn1a, Cdkn2b, Cpt1a, Egr1, Egr2, Fndc3b, Fos, Fosl2*
IKBKB	−0.44	kinase	2.16	9.56 × 10^−4^	*Bmp2, Cdkn1a, Cebpb, Egr1, Fos, Hla-A, Mt1, Mt2, Ogn, S100a4*
HGF	1.12	growth factor	2.06	3.51 × 10^−3^	*Bmp2, Calcrl, Cdc20, Cdkn1a, Cdkn2b, Crlf1, Cry1, Dok5,* *Egr1, Emp2*
CXCL12	0.36	cytokine	2.05	1.41 × 10^−6^	*Avp, Bcl3, Bmp2, Egfr, Egr1, Fos, Itgax, Lcp2, Mmp11, Nr4a1*
IL3		cytokine	2.01	1.17 × 10^−6^	*Bmp2, Ccn3, Cdkn1a, Cpt1a, Egr1, Egr2, Fcer2, Fcgr2a, Fos, Fosl2*
IKBKG	−0.42	kinase	1.97	3.25 × 10^−4^	*Bmp2, Cebpb, Fcer2, Fos, Hla-A, Mt1, Mt2, Ogn*
NTRK1	0.38	kinase	1.96	1.88 × 10^−4^	*Cdkn1a, Egr1, Fos, Nr4a1*

## Data Availability

The RNA sequencing data discussed in this publication have been deposited in NCBI’s Gene Expression Omnibus [115] and are accessible through GEO Series accession number GSE202012 (https://www.ncbi.nlm.nih.gov/geo/query/acc.cgi?acc=GSE202012, accessed on 23 May 2022).

## Supplementary Materials

The following supporting information can be downloaded at: https://www.mdpi.com/article/10.3390/cells11111784/s1, Supplementary File S1: IPA input list, Supplementary File S2: IPA output − RGC − OHT vs. SHAM. Supplementary File S3: IPA output − COLLICULUS − OHT + DREADD vs. OHT. Supplementary File S4: IPA output − RGC − OHT + DREADD vs. OHT. Supplementary File S5: Shared DEGs between OHT vs. SHAM and OHT + DREADD vs. OHT. Supplementary File S6: Metric of in vivo data.
