# Supplementary material for: Chronic Chemogenetic Activation of the Superior Colliculus in Glaucomatous Mice: Local and Retrograde Molecular Signature
Source: Cells. 2022 May 29;11(11):1784. doi: 10.3390/cells11111784 (PMC9179903; doi:10.3390/cells11111784)
Supplement: Supplementary file 1 [file cells-11-01784-s001.zip › Supplementary File 6 - Metrics of in vivo data.pdf]

cells

MDPI

Article

Chronic Chemogenetic Activation of the Superior Colliculus in Glaucomatous Mice: Local and Retrograde Molecular Signature

Marie Claes [1], Emiel Geeraerts [1], Stéphane Plaisance [2], Stephanie Mentens [1,3,4], Chris Van den Haute [5,6], Lies De Groef [3], Lut Arckens [4] and Lieve Moons [1,*]

[1] Neural Circuit Development and Regeneration Research Group, Department of Biology, KU Leuven, Leuven Brain Institute, 3000 Leuven, Belgium; marie.claes@kuleuven.be (M.C.); emiel_geeraerts@hotmail.com (E.G.); stephanie.mentens@kuleuven.be (S.M.)
[2] VIB Nucleomics core, 3000 Leuven, Belgium; stephane.plaisance@vib.be
[3] Cellular Communication and Neurodegeneration Research Group, Department of Biology, KU Leuven, Leuven Brain Institute, 3000 Leuven, Belgium; lies.degroef@kuleuven.be
[4] Neuroplasticity and Neuroproteomics Research Group, Department of Biology, KU Leuven, Leuven Brain Institute, 3000 Leuven, Belgium; lut.arckens@kuleuven.be
[5] Neurobiology and Gene Therapy Research Group, Department of Neurosciences, KU Leuven, Leuven Brain Institute, 3000 Leuven, Belgium; chris.vandenhaute@kuleuven.be
[6] KU Leuven Viral Vector Core, 3000 Leuven, Belgium
* Correspondence: lieve.moons@kuleuven.be

Supplementary information

Table S1. Mean values ± SEM for the different techniques used to probe glaucomatous induction (i.e. IOP and anterior chamber depth) and neuroprotection (i.e. pSTR recordings), corresponding to Figure 9a-c.

**Experimental group**	**Mean ± SEM**
IOP (area under the curve)	
SHAM	26.84 ± 0.53
DREADD	27.36 ± 0.45
OHT	34.97 ± 0.93
OHT + DREADD	33.68 ± 1.00
Anterior chamber depth (µm)	
SHAM	388.87 ± 2.48
DREADD	385.11 ± 3.28
OHT	472.22 ± 9.91
OHT + DREADD	472.04 ± 10.62
pSTR (µV)	
SHAM	160.79 ± 5.61
DREADD	170.74 ± 5.22
OHT	129.90 ± 5.94
OHT + DREADD	121.83 ± 4.63

Table S2. Estimation statistics (Hedges' g values with corresponding confidence intervals) for the different techniques used to probe glaucomatous induction (i.e. IOP and anterior chamber depth) and neuroprotection (i.e. pSTR recordings), corresponding to Figure 9a-c.

Comparison	Unpaired Hedges' g	95% Confidence interval
	IOP	
SHAM VS. DREADD	0.18	[-0.31, 0.67]
SHAM VS. OHT	1.75	[1.24, 2.23]
SHAM VS. OHT + DREADD	1.42	[0.92, 1.87]
	ANTERIOR CHAMBER DEPTH	
SHAM VS. DREADD	-0.22	[-0.74, 0.26]
SHAM VS. OHT	1.87	[1.45, 2.28]
SHAM VS. OHT + DREADD	1.78	[1.28, 2.37]
	pSTR	
SHAM VS. DREADD	0.32	[-0.16, 0.83]
SHAM VS. OHT	-0.92	[-1.4, -0.43]
SHAM VS. OHT + DREADD	-1.32	[-1.8, -0.81]
